# The AI-Driven Healthcare Value Framework—Rethinking Traditional Care Models in the Age of Automation

**DOI:** 10.3390/healthcare14152423

**Published:** 2026-08-06

**Authors:** Abdullah H. Alsharif

**Affiliations:** Management Information Systems Department, College of Business Administration, Taibah University, Yanbu, Saudi Arabia; alsharifa@taibahu.edu.sa

**Keywords:** AI-Augmented Healthcare, value-based care, algorithmic fairness, healthcare sustainability, patient-centered care, healthcare governance

## Abstract

**Highlights:**

**What are the main findings?**

- This study introduces a comprehensive value framework tailored to artificial intelligence in healthcare, extending evaluation beyond narrow cost–outcome ratios.
- The framework highlights patient, provider, organizational, and system-level impacts as essential dimensions of healthcare value.
- It shows that conventional healthcare value models often fail to capture equity, trust, explainability, and environmental sustainability when AI and other digital tools are used at scale.
- The proposed model organizes these considerations into a structured framework that can be applied to AI-enabled healthcare settings.

**What are the implications of the main findings?**

- The framework can help policymakers and health system leaders judge whether AI investments truly improve population health and reduce inequities.
- It provides researchers with clear dimensions for developing indicators and evaluation tools for responsible AI use in health services.
- It supports more people-centered, ethically grounded, and sustainable decision-making in healthcare innovation.
- It may also guide future implementation and assessment of AI systems in ways that balance effectiveness, fairness, and long-term system value.

**Abstract:**

**Background**: The rapid adoption of artificial intelligence (AI) in healthcare and management information systems has posed both opportunities and challenges in assessing value across clinical, operational, governance and societal dimensions. Existing healthcare value models are inadequate for the dynamic, data-rich, and ethically complex nature of AI-enabled care and thus necessitate a broader evaluative framework. **Objective**: This paper proposes an AI-Augmented Healthcare Value Framework (AI-HVF) designed as a multidimensional evaluative lens for assessing value in AI-enabled healthcare across structural, process, outcome, cost, and governance domains. **Methods**: A narrative, theory-driven literature review was undertaken of healthcare quality frameworks, value-based healthcare, digital health, AI in medicine and public health to identify limitations of existing models and to derive the required dimensions for an updated framework. The dimensions were synthesized into an integrated conceptual model linking structures, processes, outcomes, costs and governance in AI-enabled healthcare. **Results**: AI-HVF transforms traditional healthcare value models in a data-rich and automated care era. Structural dimensions include digital infrastructure, workforce readiness, learning health systems, and operational efficiency; process dimensions include AI-supported clinical excellence, patient experience, prevention, and explainability; and outcome dimensions go beyond traditional clinical metrics to include equity, safety, continuity, provider well-being, sustainability, and societal value. It also includes real cost accounting and has governance, ethics and trust as an overarching layer that supports accountability, transparency and fairness across all domains. **Conclusions**: AI-HVF provides a multi-dimensional framework to assess, plan and govern AI in health care at the patient, organization, and system levels. It is intended to enable retrospective evaluation and prospective implementation. The framework offers a foundation for developing future indicators, pilot testing and for comparative evaluation of ethical, equitable and sustainable AI integration in healthcare.

## 1. Introduction

Healthcare value is a multidimensional concept increasingly central to health systems globally. At its simplest, “value in healthcare” refers to the health outcomes achieved per unit of cost expended [1]. But this definition only scratches the surface. More comprehensive conceptions incorporate patient-centered outcomes, safety, equity, patient and provider experience, population health, and sustainable use of resources [2,3]. The term also involves satisfaction of both expressed and latent health needs in an equitable, efficient and timely manner, with minimal waste and respect for social value [4], for example, identifying undiagnosed depression in primary care before the patient explicitly reports symptoms. Value-based health care (VBHC) aims to shift systems from volume (fee-for-service, number of interventions) toward value (outcomes that matter to patients relative to costs) [3,5]. It is pivotal for effective, safe, and patient-centered healthcare, emphasizing improvements in quality, safety, and satisfaction for both individuals and populations. Patient-centered care, as a cornerstone of value, requires understanding and honoring patient preferences, meaningful communication, careful coordination, and ethical responsibility in all care processes [6]. These principles are now widely adopted, underpinning numerous frameworks that guide contemporary service delivery, driving advances in population health and clinical excellence [7]. However, the definition and operationalization of value remain dynamic, evolving alongside scientific progress, changing patient expectations, and broader sociotechnical trends [8]. Although VBHC has been influential in shifting attention toward outcomes that matter to patients, it has also been critiqued for underemphasizing equity and for framing value in ways that may insufficiently capture patient autonomy, preferences, and shared decision-making within diverse care contexts [2,3].

Despite increasing investment, the healthcare systems are struggling to deliver consistent value. Costs are continuously rising, yet many services remain restricted or underprovided, while others are overused. Standards of care often lag behind best practices, and errors in diagnosis and treatment remain common. Significant variations in quality and cost exist not only across providers but also across geographic areas, reflecting inefficiencies and inequities. Moreover, innovation is resisted, and best practices spread slowly, reinforcing systemic stagnation. Collectively, these issues indicate that traditional market competition is failing to drive improvement in healthcare outcomes and efficiency [1]. Recent literature [9,10,11,12] suggests that traditional market competition has limited ability to improve healthcare performance in fragmented delivery and financing environments. Fragmentation across providers, payers and administrative systems can undermine continuity of care, increase transaction costs and perpetuate inefficiencies, making coordination more difficult even when competitive incentives exist. At the same time, evidence from payment and delivery system reforms shows that moving away from fee-for-service incentives can have a positive impact on selected quality and cost outcomes, but impacts are often inconsistent and insufficient to address more fundamental structural issues when reforms are adopted in partially fragmented systems. These findings suggest that competition alone is unlikely to create sustained value without broader system integration, payment alignment and governance reform.

However, the rise of artificial intelligence (AI) is rapidly reshaping healthcare, catalyzing a shift in how value is created, measured, and delivered [13,14]. AI’s ability to automate diagnosis, optimize treatment planning, and personalize care pathways signals remarkable progress in efficiency, accuracy, and outcomes [15,16]. For instance, machine learning algorithms have demonstrated superior performance to traditional clinical decision-support systems in multiple diagnostic domains [17,18]. Deep learning and predictive analytics allow early detection and intervention, enhancing delivery and resource utilization [8,19]. However, AI’s disruption also carries important implications for workforce training and clinical decision-making authority, as clinicians increasingly require digital literacy, AI-specific competencies, and governance awareness to interpret algorithmic outputs critically while retaining meaningful professional oversight over patient care decisions.

Yet, this disruption also highlights significant gaps in how legacy value frameworks account for AI-enabled change [19]. Traditional models often focus on static measures of quality, safety, and satisfaction without engaging with the real-time, data-rich, and continuously adaptive nature of AI-driven systems [20]. Modern care increasingly relies on AI tools that automate complex tasks, integrate multimodal data, and support individualized patient journeys, fundamentally altering underlying assumptions about provider roles, patient engagement, and risk management [21,22].

Current healthcare value models are limited in their ability to capture the complexity of AI-infused care [17,19,23]. Most frameworks—such as value-based care, patient-centered medical homes, and accountable care organizations—were designed for human-centric, static care environments where quality and safety are measured retrospectively and personalization is constrained by provider workload and subjectivity [1,24]. In contrast, AI technologies operate dynamically, learn continuously, and often render decisions via non-interpretable mechanisms [20,21].

A critical weakness is the lack of robust evaluation criteria that reflect both the technological and social dimensions of AI deployment: integration into clinical workflows, stakeholder trust, transparency, ethical considerations, and real-world impact [8,19,25]. Furthermore, most legacy models miss the challenges posed by interpretability and generalizability, especially as AI systems may underperform in diverse or underrepresented populations [20,22]. The risk of bias in diagnostics or exclusion in personalized medicine compounds existing inequities, calling for frameworks able to scrutinize algorithmic fairness and ensure inclusive, safe application [16,21].

Personalization, once conceptualized as attentive human care, is now mediated by sophisticated prediction engines and automated treatment suggestions—often with minimal human oversight [15,16,18]. Legacy metrics for satisfaction and safety thus fail to address privacy risks, explainability, and accountability in AI-augmented medicine [8,19]. To remain meaningful and effective, healthcare value frameworks must become multidimensional, agile, and responsive to rapid technological change.

To address the deficits of existing models, this research proposes a novel AI-Driven Healthcare Value Framework designed to integrate AI technologies into established healthcare value paradigms. This framework seeks to expand the assessment of value along several new dimensions—algorithmic transparency, adaptive personalization, stakeholder trust, risk management, ethical oversight, and sociotechnical integration [19,25]. By foregrounding both patient and provider needs in the age of automation and personalization, this framework aims to restore patient-centeredness, sharpen safety protocols, and elevate quality standards in AI-enabled environments. The framework’s development draws on extensive interdisciplinary literature from health informatics, medical AI, and patient-centered care, critically synthesizing current research to articulate actionable criteria and evaluation pathways [19,25].

## 2. Methods

This study used a narrative, theory-driven literature review to develop the AI-Augmented Healthcare Value Framework (AI-HVF). The review was designed to identify, interpret, and conceptually synthesize literature on healthcare value, artificial intelligence in healthcare, digital health systems, and governance-related dimensions relevant to AI-enabled care. The aim was not to estimate pooled effects, but to build an integrative framework capable of capturing structural, process, outcome, cost, ethical, and societal dimensions of value in AI-enabled healthcare.

### 2.1. Search Strategy

A structured literature search was conducted across major academic databases relevant to healthcare, informatics, and management, including PubMed/MEDLINE, Scopus, Web of Science, and Google Scholar. The search focused on English-language literature published within a defined contemporary time frame relevant to the emergence of AI-enabled healthcare applications. Search strings combined terms related to healthcare value and AI, including “artificial intelligence,” “machine learning,” “digital health,” “healthcare value,” “value-based healthcare,” “patient-centered care,” “healthcare quality,” “algorithmic fairness,” “explainability,” “trust,” “equity,” “governance,” and “sustainability.” Boolean operators such as AND and OR were used to combine concepts, and backward citation searching of key papers was also undertaken to identify additional relevant sources.

### 2.2. Eligibility Criteria

Sources were included if they met one or more of the following criteria: (1) discussed healthcare value frameworks, quality models, or patient-centered care concepts; (2) examined AI, machine learning, or digital health applications in healthcare delivery, management, or evaluation; (3) addressed governance, ethics, explainability, fairness, trust, sustainability, or implementation issues related to AI in healthcare; or (4) provided conceptual or empirical insights relevant to framework development. Sources were excluded if they were unrelated to healthcare, did not contribute to value assessment or AI-enabled care, were duplicate records, or were purely technical papers with no clear implication for healthcare value, evaluation, or governance.

### 2.3. Selection Process

Titles and abstracts were screened for relevance, followed by full-text review of potentially eligible papers. Selection was guided by conceptual relevance rather than strict effect-based evidence hierarchy, because the purpose of the review was framework construction. Through this process, 58 studies and conceptual sources were synthesized. These sources represented a combination of foundational healthcare value literature, AI-in-healthcare studies, digital health and informatics research, and governance- and ethics-oriented publications that informed the final framework dimensions.

### 2.4. Quality and Relevance Appraisal

Because this was a narrative, theory-driven review rather than a systematic review or meta-analysis, formal risk-of-bias scoring was not applied. Instead, sources were appraised for conceptual relevance, methodological clarity, disciplinary credibility, and contribution to framework development. Greater weight was given to foundational theoretical models, influential review papers, empirical healthcare AI studies with clear methodological reporting, and publications directly addressing dimensions such as equity, trust, explainability, sustainability, and implementation. This approach was used to maintain conceptual rigor while allowing inclusion of both empirical and theoretical literature necessary for model development.

### 2.5. Synthesis of Dimensions

The synthesis followed an iterative deductive–inductive approach. Deductively, established healthcare value models—particularly Donabedian’s Structure–Process–Outcome model, Porter’s Value-Based Healthcare framework, and the Quadruple Aim—were used as anchor frameworks for organizing value-related concepts. Inductively, the included literature was examined for recurring AI-specific dimensions that were insufficiently addressed in traditional models, such as digital infrastructure, workforce readiness, algorithmic fairness, explainability, trust, governance, sustainability, and provider well-being. These dimensions were compared across sources, grouped into conceptually related domains, and refined through repeated review until a coherent multidimensional framework emerged.

### 2.6. Framework Development

The AI-HVF is a conceptual framework developed from narrative theory-driven literature synthesis and has not yet been empirically implemented or validated in a real-world healthcare setting. The final AI-HVF was developed by integrating recurring dimensions across the reviewed literature into a single conceptual structure linking structural foundations, care processes, outcomes, cost considerations, and an overarching governance layer. Dimensions were retained when they were conceptually distinct, repeatedly supported across the literature, and essential to evaluating value in AI-enabled healthcare systems. This process resulted in a framework intended for both retrospective evaluation and prospective planning of AI integration in healthcare settings.

## 3. Literature Review

### 3.1. AI’s Impact on Healthcare Value Dimensions

The rise of AI in healthcare has led to significant advancements, particularly in predictive analytics, generative AI, and automated patient management. Predictive analytics harness large datasets and machine learning to enhance diagnostics and disease prevention efforts by identifying risk factors and patterns that evade traditional clinical assessment [26,27]. While predictive models improve early detection and patient stratification, concerns regarding data bias, model transparency, and equitable implementation remain pressing [28,29]. Taken together, these developments show that AI in healthcare should be understood not only as a technological advance, but as a source of new value opportunities and new ethical, organizational, and equity-related risks.

Generative AI has demonstrated potential in automating medical documentation, improving the accuracy and speed of imaging analysis, and facilitating patient–provider communication [23,30]. These technologies reduce administrative burdens and enhance diagnostic workflows, yet they also introduce challenges related to model explainability, risks of misinformation, and safeguarding patient privacy [31,32]. Ethical considerations around consent and accountability for AI-generated medical content are increasingly critical in this evolving landscape [33].

Furthermore, AI-driven automated triage and personalized treatment planning are revolutionizing clinical pathways by optimizing resource allocation and tailoring care to individual patient profiles [34,35]. AI-powered patient engagement platforms offer continuous, adaptive support but raise concerns about the depersonalization of care and the digital divide that may exacerbate health inequities [36,37]. Beyond these applications, AI is increasingly utilized in operational efficiency—such as resource allocation, scheduling, and supply chain optimization—reducing costs but raising questions of workforce displacement [38].

Despite these promising developments, critical limitations persist. Algorithmic biases, interpretability challenges, and regulatory gaps require rigorous oversight to ensure AI tools contribute positively without compromising patient safety or ethical standards [19,21]. The literature underscores the necessity for interdisciplinary frameworks that balance innovation with trust, equity, and human-centered values in healthcare.

In this context, healthcare management information systems play a pivotal role as the digital backbone for AI-enabled transformation. Rather than referring to MIS in a broad organizational sense, this paper focuses on their informatics function in integrating clinical and operational data, supporting interoperable workflows, enabling decision support, and facilitating governance for AI systems. These capabilities are essential for translating AI outputs into actionable value, because they connect algorithmic tools with real-world care processes, accountability mechanisms, and continuous learning. In this sense, MIS are not merely administrative support systems but core infrastructure for operationalizing AI-HVF in healthcare.

### 3.2. Domain-Specific Impacts of AI on Healthcare Value

Healthcare value is a multifaceted concept encompassing diverse dimensions vital for delivering effective, equitable, and sustainable care. AI is reshaping foundational dimensions of healthcare value—health outcomes, patient experience, safety, efficiency, equity, coordination, personalization, technical quality, governance, societal value, sustainability, prevention, continuity and trust—but its effects are heterogeneous, context-dependent and sometimes contradictory. Evidence indicates substantial potential to improve measurable health outcomes through enhanced diagnostic accuracy and predictive risk stratification: machine-learning models can detect disease signals earlier than conventional approaches, improving timeliness of intervention [21,39]. However, gains in controlled studies do not always translate to real-world outcome improvements, because model performance degrades across settings and populations [40]. Thus, AI’s promise for outcomes is real but conditional on data quality, external validation and integration into care pathways. Nevertheless, the use of AI in healthcare is constrained by overfitting to training data and a lack of robust external validation, limiting the generalizability, reproducibility, and clinical reliability of model performance across settings. Overall, it has to be understood that AI does not affect a single aspect of healthcare value in isolation, but reshapes multiple interdependent dimensions that must be evaluated together.

Health outcomes serve as foundational indicators of healthcare value, focusing on measurable gains such as symptom relief, disease control, and longevity [41,42]. However, traditional outcome measurement often neglects heterogeneity in patient populations and fails to capture nuanced or long-term benefits. AI technology enhances outcome assessment through predictive analytics and real-time data integration, allowing for earlier intervention and personalized prognosis [14]. Yet, reliance on algorithmic outputs necessitates scrutiny regarding data quality and bias, which may skew outcomes for marginalized groups [43]. Thus, outcome improvement in AI-enabled care depends not only on technical performance, but also on fairness, generalizability, and implementation quality.

Patient experience and satisfaction encompass subjective dimensions like comfort, respect, and communication quality, critical to adherence and trust [2,39]. AI-driven conversational agents and digital platforms can augment patient engagement by offering tailored information and 24/7 support [44]. Studies [45,46] on explainable AI (XAI) suggest that transparent model explanations can promote clinician trust, improve interpretability, and support accountable AI-assisted decision-making in healthcare. Nonetheless, risks of depersonalization and reduced human contact challenge the preservation of empathy and holistic understanding that underpin true patient-centeredness. These dimensions show that the value of AI depends as much on how it is integrated into care relationships and systems as on what the algorithm can technically do.

Equity and access address fair distribution of healthcare resources and services. Structural inequities often perpetuate disparities despite policy efforts [39,41]. AI holds promise in identifying underserved populations and mitigating biases but may inadvertently exacerbate inequities if datasets lack representativeness or if deployment favors resource-rich settings [22]. Algorithmic bias and systemic inequity are related but distinct. Algorithmic bias refers to unfairness introduced by the AI model itself, such as bias from training data or model design, whereas systemic inequity refers to broader structural disparities in access, resources, and care delivery within the health system. In the discussion of equity, both should be addressed because biased algorithms can reinforce existing inequities, but improving the model alone will not resolve underlying social and organizational injustice. This distinction is consistent with equity-monitoring approaches such as the WHO Health Equity Monitor and with digital health equity frameworks that emphasize representation, access, implementation context, and distributional effects across populations [47,48]. Care coordination seeks seamless integration across providers and settings to avoid fragmentation. AI supports coordination via interoperable electronic health records and predictive analytics that alert care teams to patient needs [28]. However, interoperability challenges and data silos persist as barriers. Interoperability remains a significant implementation challenge, because AI systems must be integrated with legacy health information infrastructure; although HL7 FHIR provides a standardized approach to data exchange, heterogeneity in system design, incomplete adoption, and challenges in mapping data across platforms continue to impede seamless integration [49,50].

Personalization or patient-centeredness signifies care tailored to individual goals and contexts [51]. AI enables dynamic personalization through data-driven insights, enhancing relevance of care plans [52]. Yet, personalization must guard against algorithm-driven standardization that risks ignoring patient preferences. Technical quality emphasizes adherence to evidence-based medicine [53]. AI may reinforce quality by providing decision support and standardizing protocols but must avoid rigidity that impairs clinical creativity. System-level governance and leadership underpin a value-driven culture and infrastructure [41,52]. AI implementation demands visionary leadership to ensure ethical use, data governance, and equitable integration. Taken together, these considerations indicate that AI can strengthen healthcare value only when personalization, technical quality, and governance evolve in parallel.

Societal value reflects healthcare’s contribution to social cohesion and participation [51]. AI’s role here is nascent, but potential exists in population health analytics supporting community-wide health strategies. Sustainability emphasizes balancing current healthcare delivery with environmental stewardship. AI can optimize supply chains and reduce waste but must be environmentally conscious in energy-intensive data processing. Wellness and prevention focus on proactive health promotion. AI-powered monitoring and behavior change interventions can foster healthier lifestyles, reducing disease burden. Continuity of care ensures consistent, coordinated services over time, essential for chronic condition management [54]. AI facilitates continuity through integrated data systems but risks fragmenting care if interoperability is insufficient. Trust is another dimension that fundamentally enables acceptance of care and technology [2]. AI’s opacity and potential biases threaten trust, necessitating transparency, explainability, and patient involvement to maintain confidence. Accordingly, any meaningful evaluation of AI in healthcare must extend beyond clinical performance to include broader social, environmental, and relational consequences.

Overall, AI impacts all healthcare value dimensions, offering transformative potential tempered by significant ethical, social, and practical challenges. The integration of AI must be guided by rigorous governance and a commitment to equity and patient-centered values to genuinely enhance healthcare value.

### 3.3. Traditional Healthcare Value Models

Traditional healthcare value models have long guided efforts to enhance care quality, efficiency, and patient outcomes. Among the most influential are Donabedian’s Structure–Process–Outcome (SPO) model, Porter’s Value-Based Healthcare (VBHC) framework, and the Quadruple Aim framework. While these models have shaped healthcare improvement, their limitations are increasingly exposed in the context of rapid AI-driven automation and digital transformation. This makes the reassessment of traditional value models necessary rather than optional in the context of AI-enabled healthcare.

Donabedian’s triadic SPO model conceptualizes healthcare quality as the interplay of structural factors (e.g., facilities, workforce), care processes (e.g., diagnosis, treatment), and outcomes (e.g., morbidity, mortality) [55]. This framework emphasizes a linear cause–effect relationship where good structures enable effective processes and desirable outcomes. Despite its foundational role in quality assessment, the SPO model is inherently static, focusing on observable, retrospective measures that inadequately capture the dynamic, data-rich realities of AI-enabled care [56]. AI’s integration facilitates continuous, real-time monitoring and feedback loops that challenge the traditional linearity and segmentation of structure, process, and outcome. Furthermore, Donabedian’s model offers limited guidance on integrating complex data streams, algorithmic decision-making, or the socio-technical aspects of AI–human collaboration. Donabedian’s structure dimension can be adapted for AI-enabled care by explicitly incorporating algorithmic infrastructure, data governance, interoperability capacity, workforce digital readiness, and regulatory oversight as core structural elements, as reflected in recent digital health applications of the model [57]. Donabedian’s model remains foundational, but its continued usefulness in AI-enabled care depends on substantial expansion of what counts as structure, process, and outcome.

Porter’s VBHC model redefines value as health outcomes achieved per dollar spent, spotlighting measurable outcomes relative to costs along the full care cycle [4]. This model incentivizes outcome measurement and cost containment, promoting patient-centeredness by focusing on what matters to patients. While influential, VBHC remains constrained by reliance on traditional outcome metrics and episodic evaluation [3]. In AI’s age, the volume, velocity, and variety of health data surpass conventionally measurable outcomes, demanding new metrics capturing AI’s impact on predictive accuracy, workflow augmentation, and personalized interventions. Moreover, VBHC does not systematically address the ethical, transparency, and trust issues intrinsic to AI deployment, nor does it explicitly incorporate patient engagement through digital platforms, which profoundly influence perceived value.

In addition, health economists and ethicists have argued that VBHC can become too narrow when reduced to a cost-outcome equation, because this framing may underemphasize allocative fairness, patient autonomy, and broader social value. Empirical reviews also suggest that VBHC implementation often remains dominated by provider-defined process, cost, and clinical outcome measures rather than patient-centred indicators, raising concerns that the model may not fully capture what patients value most [58]. Therefore, while VBHC remains influential, its cost-outcome logic is too narrow to fully capture value in AI-mediated healthcare.

The Quadruple Aim framework extends the Triple Aim by adding provider well-being to goals of improving population health, enhancing patient experience, and reducing burnout [59]. This holistic approach recognizes provider burnout as a critical factor influencing healthcare quality. However, its broad goals lack specificity on operationalizing value in AI-driven contexts. AI-enabled automation reshapes provider roles, potentially alleviating burnout but also risking deskilling or alienation if poorly implemented. The Quadruple Aim does not explicitly consider AI’s role in reshaping teamwork, cognitive workload, or ethical challenges from algorithmic opacity and bias.

For this reason, the Quadruple Aim offers an important foundation but remains incomplete for assessing AI-related transformations in care delivery.

Collectively, these traditional models fall behind in the era of AI automation by underemphasizing several critical dimensions (see Table 1). First, the models do not adequately factor in the continuous learning and adaptation of AI systems that can iteratively improve prediction and treatment beyond static protocols. Second, they insufficiently capture multidimensional value drivers—including algorithmic fairness, data security, patient trust in AI decisions, and explainability—core to ethical AI adoption. Third, they lack integration of patient-generated health data and real-time monitoring enabled by wearables and mobile health technology, which redefine care boundaries. Fourth, the human–AI interaction spectrum and its sociotechnical implications remain largely unaddressed, yet these are pivotal for sustaining clinician engagement and shared decision-making. Finally, they do not incorporate the potential environmental impacts of digital health infrastructure or sustainability considerations increasingly relevant in value assessments.

The integration of AI within healthcare underscores an urgent need for robust MIS capable of supporting adaptive, feedback-driven frameworks. Traditional models, with isolated data silos and retrospective analysis, fail to capture the dynamic learning cycles and real-time decision-making characteristic of AI-powered systems. MIS not only bridge these gaps by enabling continuous data capture, interoperability, and analytics but also enhance algorithmic accountability and transparency, which are crucial for equitable and ethical application of AI in health services. Taken together, these limitations show why existing frameworks cannot simply be reused unchanged in the AI era.

In the AI-enabled healthcare era, traditional value models require expansion because they do not fully capture the continuous learning, adaptive decision-making, and sociotechnical complexity introduced by artificial intelligence. AI-HVF addresses this gap by explicitly incorporating digital infrastructure, data governance, explainability, fairness, trust, and workflow integration as value-relevant dimensions. In this way, the framework transforms conventional value assessment by extending structure beyond physical and human resources to include algorithmic and informational infrastructure, extending process beyond routine care delivery to include AI-supported decision-making and personalization, and extending outcomes beyond clinical endpoints to include equity, sustainability, provider well-being, and societal value.

## 4. AI-Augmented Healthcare Value Framework

The evolution of healthcare value frameworks reflects changing paradigms in medicine. Donabedian’s Structure–Process–Outcome model, Porter’s Value-Based Healthcare framework, and the Quadruple Aim each provided conceptual clarity for their time, yet they remain tied to the assumptions of analogue healthcare systems. As highlighted in the introduction to this study, these traditional models are constrained by a linear and static understanding of value generation, an overly narrow focus on outcomes defined solely in clinical terms, insufficient attention to equity and inclusion, inadequate recognition of hidden costs, weak provisions for governance, and the omission of sustainability. The rise of AI amplifies these gaps, since algorithmic systems are dynamic, feedback-driven, and introduce new risks as well as new opportunities. To address such gaps, Oyekunle et al. [60] highlights the necessity of new value models that integrate algorithmic fairness, explainability, and patient engagement through digital platforms. Similarly, Rajpurkar et al. [61] advocate for adaptive frameworks that evaluate AI tools across technical performance, clinical impact, and societal implications, moving beyond static measures. For this reason, AI-HVF is proposed not as a minor extension of prior models, but as a broader reconceptualization of healthcare value for the AI era.

**Figure 1:**
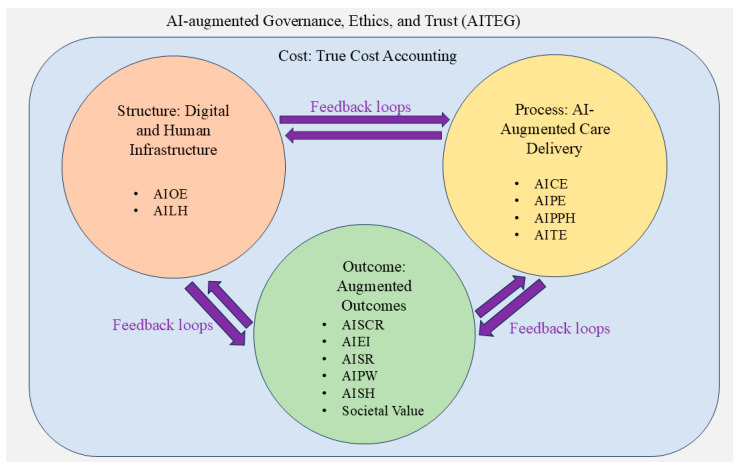
AI-Augmented Healthcare Value Framework.

The AI-Augmented Healthcare Value Framework (AI-HVF) is therefore proposed as an integrative and adaptive framework designed to capture the complexity of AI-enabled health systems. This framework is intended for both retrospective evaluation of existing AI-enabled healthcare interventions and prospective planning of future implementations, thereby supporting assessment, design, and continuous improvement across the care cycle. It does so by reimagining the classical categories of structure, process, outcome, and cost, embedding them within a governance shell (refers to the external layer of principles and oversight mechanisms that ensures AI systems are safe, transparent, fair, and accountable across the framework, in line with the OECD AI Principles on trustworthy AI), and by introducing novel dimensions that are critical for an era of automation. Relevant bioethics frameworks and IRB processes should also inform this governance layer, particularly for AI research involving consent, privacy, bias mitigation, data security, and algorithmic transparency. The inclusion of these dimensions is not an embellishment but a necessity. Excluding them would replicate the very gaps that undermine existing models. If clinical excellence is measured without algorithmic accountability, diagnostic gains may mask systemic risks. If equity is excluded, biased algorithms may exacerbate disparities. If sustainability is ignored, healthcare may achieve local improvements at the expense of global ecological stability. If provider well-being is overlooked, efficiency gains may be purchased through clinician exhaustion. In short, the new dimensions transform healthcare value from a narrow economic equation into a multidimensional, ethically grounded, and sustainability-oriented construct. Sustainability is considered as a core dimension of AI-enabled healthcare value because digital health interventions also carry environmental costs, including energy use, device lifecycles, data storage, and model training. Recent reviews recommend evaluating the carbon footprint of digital health solutions through environmental impact assessment and life-cycle approaches, while carbon accounting tools can help quantify emissions and support greener implementation choices [62,63,64]. These novel dimensions ensure that AI delivers not only efficiency but also fairness, trust, and planetary responsibility. Figure 1 illustrates the conceptual design of the framework. Viewed together, these features position AI-HVF as a framework for judging whether AI creates value responsibly rather than merely efficiently.

At the operational core of the AI-Augmented Healthcare Value Framework lies a firm reliance on Management Information Systems to support digital infrastructure, data governance, and workflow automation. Advanced MIS architectures are instrumental in orchestrating real-time data collection, supporting continuous learning health systems, and ensuring that feedback from AI-driven outcomes informs further structural enhancements. This symbiotic relationship guarantees that the framework’s adaptive and cybernetic characteristics are achieved in practice, promoting sustainability, resilience, and system-wide value. Thus, MIS is not peripheral to AI-HVF but central to making its adaptive and evaluative logic operational in practice.

### 4.1. Components

AI-HVF is informed by existing governance and ethics frameworks, particularly the WHO guidance on ethics and governance of artificial intelligence for health and the OECD AI Principles. The WHO guidance emphasizes human autonomy, well-being, transparency, accountability, inclusiveness, equity, responsiveness, and sustainability, while the OECD AI Principles stress human rights, democratic values, transparency, robustness, safety, and accountability across the AI lifecycle. AI-HVF extends these principles into a healthcare value framework by translating them into operational dimensions and evaluative indicators for clinical, organizational, and system-level use.

#### 4.1.1. Structure: Digital and Human Infrastructure

Donabedian’s original conception of structure referred to the material and human resources available within healthcare institutions. In the AI era, this interpretation is no longer sufficient. The capacity of a healthcare system to create value increasingly depends on its digital infrastructure, including interoperable electronic health records, secure cloud and edge computing environments, and machine learning pipelines. Equally important is the preparedness of the workforce, encompassing digital literacy, AI training, and new governance competencies. Structure in the AI era therefore depends on digital and human readiness as much as on traditional institutional resources.

The inclusion of AI-Enabled Operational Efficiency (AIOE) is critical here. One of the major gaps in Porter’s model is its narrow understanding of cost, which assumes that efficiency gains are achieved largely through payment reform or process redesign. AI introduces efficiency at a deeper infrastructural level by automating administrative tasks, streamlining supply chains, and optimizing scheduling systems [65]. Without incorporating AIOE into the structural layer, value assessments would underestimate AI’s transformative capacity while simultaneously ignoring the recurrent costs and risks of automation. Accordingly, operational efficiency must be treated as a structural source of value rather than merely a downstream byproduct of care delivery.

AI-Powered Learning Health Systems (AILH) is a second structural dimension that directly addresses Donabedian’s linearity problem. Structures in AI-HVF are not fixed backdrops but dynamic entities capable of self-renewal through real-world data integration. By continuously feeding outcomes back into infrastructural and organizational design, AILH creates a structural environment that is inherently adaptive [66]. This is essential because static structures cannot accommodate the rapid cycle times of AI model development, retraining, and deployment. This makes adaptive learning capacity a defining structural requirement for value creation in AI-enabled health systems.

#### 4.1.2. Process: AI-Augmented Care Delivery

The process dimension in Donabedian’s model traditionally measured compliance with guidelines or the timeliness of interventions. Such measures, while still relevant, are profoundly inadequate in the age of AI. Processes are no longer limited to the delivery of human-mediated care but increasingly involve algorithmic interactions, predictive systems, and generative models. Process quality must therefore be redefined to include not only what care is delivered, but how algorithmic systems shape its delivery.

AI-Supported Clinical Excellence (AICE) extends the definition of process quality by embedding diagnostic accuracy, risk prediction, and treatment personalization into the very fabric of care. This dimension directly addresses the gap of narrow outcome definitions, because it recognizes that algorithmic excellence itself must be measured as part of process quality. Without this recognition, traditional models would fail to capture the added value of AI systems that outperform humans in specific diagnostic tasks. In this way, clinical excellence in AI-enabled care becomes a process attribute that must be evaluated continuously rather than assumed from outcomes alone.

AI-Enhanced Patient Experience (AIPE) is equally critical. Traditional models acknowledged the importance of patient satisfaction but lacked the mechanisms to evaluate the profound ways AI reshapes communication. Conversational agents, multilingual support systems, and personalized digital interfaces redefine how patients interact with health services [67]. If not accounted for, these innovations risk undermining empathy, trust, and cultural sensitivity, thereby reinforcing inequities. AIPE ensures that processes remain anchored in patient-centered values even in technologically mediated environments. This ensures that patient experience remains a core measure of value even when care is increasingly mediated through digital and automated channels.

AI-Driven Preventive and Population Health (AIPPH) directly respond to the downstream orientation of Porter’s value equation. By focusing on disease control and survival, Porter’s model neglected upstream determinants of health. AI enables proactive identification of risk, epidemic forecasting, and remote monitoring, thereby shifting the focus from treatment to prevention [68]. Including AIPPH in the process layer ensures that the framework aligns with public health imperatives and not just individual episodes of care. By doing so, the framework expands value from reactive treatment toward proactive and population-oriented care.

Finally, AI-Enabled Transparency and Explainability (AITE) addresses one of the most pressing gaps: governance. Traditional models assumed that process quality was transparent to both patients and providers. In AI-mediated processes, however, algorithmic opacity creates a “black box” problem [69]. AITE ensures that interpretability is embedded as a process value, providing clinicians and patients with rationales for AI-generated recommendations. Without this dimension, adoption risks stalling in the face of distrust and legal uncertainty. Transparency and explainability are therefore not secondary technical features, but essential process conditions for trust and responsible use.

Explainability is an important feature of AI-enabled healthcare because it can support accountability, user understanding, and appropriate oversight. However, explainability does not automatically outweigh predictive performance, and the optimal balance between interpretability and accuracy may vary by clinical use case, risk level, and decision context. In some applications, a highly accurate but less interpretable model may be justified if it is externally validated, continuously monitored, and used within appropriate governance safeguards. AI-HVF therefore treats explainability as a valued process dimension, not as a universal replacement for clinical utility or model performance.

The governance layer is further supported by recent AI governance scholarship emphasizing multi-layered oversight, digital sovereignty, and accountability across policy, institutional, technical, and operational levels. This perspective reinforces the need for AI-HVF to treat governance not as a peripheral safeguard but as a core evaluative layer for ensuring responsible AI deployment in healthcare [70]. AI-enabled care processes should also be informed by participatory design and co-creation with patients, clinicians, and other stakeholders to ensure that digital tools remain usable, context-sensitive, and aligned with real care needs.

The governance layer should also explicitly address data security, digital sovereignty, and cybersecurity risk. Recent AI governance literature emphasizes that responsible AI deployment requires layered oversight across policy, institutional, technical, and operational levels, alongside controls for privacy, security, incident response, and accountability. In healthcare, these issues are especially critical because AI systems may expose sensitive health data, create vendor dependency, enable unauthorized access, or introduce new attack surfaces such as data poisoning and adversarial manipulation. AI-HVF therefore treats AI risk governance not only as an ethical concern but also as a structural requirement for value creation and safe deployment.

#### 4.1.3. Outcome: Augmented Outcomes

The outcome dimension has historically centered on survival, symptom relief, and functional status. While these remain fundamental, they are insufficient in the AI context. Outcomes now include algorithmic performance, trust, equity, and environmental sustainability. AI-HVF redefines outcomes as multidimensional, reflecting both human and machine contributions to health value. Outcome assessment must therefore capture not only what happens to patients, but also how AI influences fairness, safety, trust, and long-term system effects. Trust is treated primarily as an outcome because it reflects whether patients, clinicians, and organizations accept and rely on AI-enabled care; however, it is also shaped by structural readiness and process quality, including transparency, explainability, governance, and user engagement. In this framework, fairness is necessary but not sufficient for equity: a model may be statistically fair yet still produce inequitable access or exclude underserved groups from meaningful benefit.

AI-Supported Care Continuity and Retention (AISCR) respond to a long-standing gap in traditional frameworks: the fragmentation of care. Episodic outcome measures often ignored the importance of follow-up, adherence, and long-term engagement. AI tools for remote monitoring, post-discharge reminders, and chronic disease management ensure that continuity is valued alongside acute outcomes [71,72]. This reframes continuity as a central outcome of value rather than a secondary feature of service organization.

AI-Enabled Equity and Inclusion (AIEI) addresses equity blindness, perhaps the most critical limitation of earlier models. Algorithms trained on biased data risk amplifying disparities, yet neither Donabedian nor Porter incorporated equity explicitly. AIEI mandates fairness audits, inclusion of diverse datasets, and culturally sensitive algorithm design. It makes equity not an afterthought but a central determinant of value [73]. Equity is therefore treated in AI-HVF as a direct outcome of value creation, not simply as an external ethical concern. AIEI should also include access metrics indicating who is actually reached or served by AI interventions, participatory design indicators showing whether affected users were involved in development and refinement, and community engagement measures capturing the extent to which local populations shape implementation and oversight.

AI-Integrated Safety and Reliability (AISR) expand Donabedian’s safety dimension to include algorithmic harms. Traditional safety metrics do not capture model drift, adversarial attacks, or unintended consequences of automation. AISR ensures that AI safety is continuously monitored and integrated into outcome assessments [74]. This broadens safety from protection against clinical error to protection against algorithmic failure and unintended digital harms.

AI-Supported Provider Well-Being (AIPW) reflects the insight of the Quadruple Aim, which recognized provider burnout as a systemic risk. In the AI era, the risk is magnified: automation may reduce administrative burdens but can also generate new forms of cognitive overload and alert fatigue [75]. Including AIPW as an outcome dimension ensures that value is not achieved at the expense of clinician well-being. Including provider well-being therefore ensures that efficiency gains are not pursued at the hidden cost of workforce sustainability.

AI-Driven Sustainability and Green Health (AISH) address the environmental footprint of AI and digital health across the full technology life cycle. This includes energy consumption during model training and deployment, device manufacturing and disposal, electronic waste, data-center and cloud storage demands, and associated carbon emissions. AISH therefore captures both operational and upstream/downstream environmental impacts, ensuring that sustainability is evaluated as a systems-level outcome rather than only as a per-run efficiency metric [76]. AISH integrates planetary health into healthcare value, ensuring that short-term clinical gains do not undermine long-term ecological viability. This makes environmental sustainability an integral outcome of healthcare value in digital and AI-intensive settings.

Finally, the framework incorporates Societal Value, recognizing that healthcare’s impact extends beyond individual patients to communities. AI systems deployed in vaccination campaigns, epidemic forecasting, or public health education contribute to social resilience and connectedness, dimensions of value ignored in classical formulations [77,78]. Societal value contribution refers to the broader system-level benefit created by an AI intervention beyond direct clinical outcomes, including access improvement, reduced inequity, improved population coverage, reduced avoidable utilization, and public health impact. It may be approximated using indicators such as reach in underserved populations, avoided downstream utilization, reduced wait times, or equity-adjusted outcome gains. Societal value therefore extends the framework beyond individual care episodes to the wider public consequences of AI deployment.

#### 4.1.4. Cost: True Cost Assessment

Porter’s denominator of cost was conceived primarily in economic terms. Yet AI introduces layers of hidden and recurrent costs, from infrastructure maintenance and cybersecurity to workforce retraining and carbon emissions [79,80]. Evaluating value without acknowledging these dimensions risks producing inflated or misleading assessments. Cost in AI-HVF therefore embraces total cost of ownership (TCO), which includes not only direct expenditures but also governance, ethical oversight, opportunity costs, and environmental externalities. This expansion is critical for accountability. Without it, claims of efficiency might be based on narrow financial metrics while ignoring the ethical and ecological burdens of AI deployment. True Cost Assessment is therefore essential if AI-related value claims are to remain credible, comparable, and ethically grounded. To support practical use, AI-HVF includes at least one illustrative metric for each major dimension. For the cost dimension, TCO should be calculated as the sum of direct and indirect costs incurred over the AI system lifecycle, including acquisition or licensing, integration, data infrastructure, compute resources, staff training, cybersecurity, validation audits, monitoring, retraining, maintenance, and workflow disruption. For other dimensions, example metrics include subgroup performance gaps for fairness, patient trust scores for trust, and time saved or alert burden for workflow efficiency.

*TCO = acquisition + integration + infrastructure + training + cybersecurity + validation + monitoring + retraining + maintenance + workflow disruption*

The cost dimension in AI-HVF is intended to capture the full economic burden of AI across its life cycle rather than only upfront procurement costs. This includes compute resources, data infrastructure, integration, workforce training, validation audits, continuous monitoring, model updating, maintenance, cybersecurity, regulatory compliance, and workflow disruption. To evaluate value, these costs should be considered alongside clinical utility and patient outcomes using a cost-consequence or cost-effectiveness logic in which implementation cost is weighed against improvements in diagnostic accuracy, efficiency, safety, patient experience, and avoided downstream harm. In practice, AI-HVF is designed to support total cost of ownership analysis rather than a single-point purchase-cost estimate.

The inclusion of real cost accounting in AI-HVF reflects the need to capture not only direct implementation expenses but also the full lifecycle costs associated with AI adoption in healthcare. These include costs related to digital infrastructure, model development and maintenance, staff training, cybersecurity, interoperability, ethical review, monitoring, and ongoing governance. In addition, governance, ethics, and trust function as an overarching layer because they shape accountability, transparency, fairness, and responsible oversight across all domains of the framework. This layer ensures that AI-enabled care is assessed not only for efficiency and clinical performance, but also for its ethical acceptability, regulatory alignment, and social legitimacy.

#### 4.1.5. AI-Augmented Governance, Ethics, and Trust (AITEG)

Perhaps the most innovative departure of AI-HVF is its treatment of governance. Traditional frameworks regarded governance as external to the value equation, assuming that ethical and regulatory safeguards would operate independently. In an AI context, this assumption is untenable. Trust, fairness, and transparency are not optional add-ons; they are prerequisites for adoption [33,43]. AITEG therefore forms the outer shell of the framework, encasing all other dimensions. It ensures that every structural, processual, outcome, and cost decision is filtered through ethical oversight, fairness audits, privacy safeguards, and accountability triggers. By institutionalizing governance as a structural layer, AI-HVF acknowledges that value cannot be created in the absence of trust. In this framework, governance is not external oversight added after implementation, but a constitutive condition of healthcare value itself.

The governance shell (AITEG) influences each dimension of AI-HVF in practical ways. At the structural level, it requires secure data governance, privacy safeguards, interoperability standards, and workforce accountability mechanisms before AI tools are deployed. At the process level, it shapes how algorithms are selected, validated, monitored, and explained, ensuring that transparency, consent, and fairness are embedded in clinical workflows. At the outcome level, governance determines whether equity, safety, trust, and continuity are actively monitored through audit trails, bias detection, and adverse event reporting. At the cost level, it requires inclusion of hidden governance expenses such as ethics review, compliance, retraining, and cybersecurity. In this sense, AITEG is not only an outer boundary of the framework but also a cross-cutting control layer that influences how value is created, measured, and sustained across the system.

### 4.2. Interrelationships and Feedback Loops

A defining feature of AI-HVF is its cybernetic and adaptive design. In contrast to Donabedian’s linear model, AI-HVF emphasizes that outcomes inherently feed back into processes and structures in real time. Equity outcomes demand systematic re-engineering of data pipelines to eliminate bias and ensure inclusion. Provider well-being outcomes necessitate redesign of workflows and clinical processes to reduce administrative and cognitive burdens. Sustainability outcomes require recalibration of cost calculations to incorporate environmental impacts and energy consumption. These relationships show that value in AI-enabled healthcare is continuously produced through feedback rather than through a one-way chain of causation.

These feedback loops are not optional enhancements but constitutive features of an AI-enabled healthcare system. They reflect the fundamental reality that AI systems are continuously learning, evolving, and adapting, and that healthcare organizations must mirror this dynamism in order to remain both effective and ethical. The concentric circular embodies this interdependence, making visible the cyclical flow of influence between structure, process, outcomes, and costs. This stands in sharp contrast to the static and unidirectional arrows of earlier models, which fail to capture the iterative and self-correcting nature of AI-driven healthcare. For this reason, AI-HVF is designed as a learning framework capable of supporting continuous adjustment, accountability, and improvement.

These interrelationships can be made more explicit through several recurring feedback loops. First, poor equity outcomes may trigger dataset review, subgroup validation, and redesign of deployment protocols, which then reshape both structure and process. Second, workflow disruption or provider burden may reduce trust and adoption, which in turn weakens patient experience, continuity, and overall outcome performance unless workflows are redesigned. Third, safety events or evidence of model drift may require recalibration, stronger governance controls, and revised clinical oversight, thereby feeding outcomes back into cost, process, and structure simultaneously. In this way, AI-HVF treats outcomes not as endpoints, but as signals that continuously modify the conditions of future care.

AI-HVF is intentionally designed as a dynamic framework rather than a static checklist. Its cybernetic structure allows new AI capabilities to be incorporated by updating the relevant dimensions, metrics, and governance mechanisms as technologies evolve. For example, emerging generative, multimodal, or agentic AI systems can be assessed within existing domains such as clinical excellence, transparency, safety, equity, and provider well-being, while new indicators can be added to Table 2 without altering the framework’s core architecture. In this way, AI-HVF is intended to remain stable in its conceptual logic but flexible in its operationalization, enabling continuous refinement as AI technologies, clinical applications, and governance requirements change. The causal direction is therefore reciprocal rather than one-way: strong structures and transparent processes build trust, and higher trust in turn improves adoption, adherence, and sustained use of AI systems, which feeds back into better outcomes.

### 4.3. Operational Definitions and Indicators

To improve the practical applicability of AI-HVF, the main constructs are defined in operational terms. Real cost accounting refers to the full life-cycle cost of AI adoption, including acquisition, integration, training, workflow redesign, monitoring, recalibration, governance, cybersecurity, and disruption-related costs. Algorithmic fairness refers to comparable performance and benefit across relevant patient groups, while adaptive personalization refers to tailoring AI-supported recommendations to patient needs, preferences, and evolving clinical context. Governance shell (AITEG) refers to the cross-cutting oversight layer that ensures accountability, transparency, privacy, fairness, safety, and compliance across all dimensions.

In addition, trust is treated primarily as an outcome reflecting willingness to rely on AI-supported care, although it is shaped by structures and processes; fairness drift refers to inequities that emerge or worsen after deployment; workflow disruption refers to unintended burdens such as alert fatigue, delays, or added documentation; model recalibration and retraining refer to corrective updating when performance or fairness declines; explainability refers to the degree to which AI outputs can be made understandable to clinicians, patients, or oversight bodies; and human-in-the-loop oversight refers to the continued role of qualified professionals in reviewing, confirming, or overriding AI recommendations. These constructs can be linked to indicators such as total cost of ownership, subgroup performance gaps, patient trust scores, override rates, drift incidents, workflow burden, recalibration frequency, and explanation usefulness, thereby supporting empirical testing and implementation planning.

### 4.4. Operationalization of AI-HVF

Conceptual frameworks gain true value only when effectively operationalized. Table 2 fulfills this role by transforming abstract dimensions into tangible, measurable indicators. For each novel dimension, clear definitions, descriptions, and associated metrics have been established. These metrics range widely—for example, clinical accuracy scores for AICE, fairness audit outcomes for AIEI, energy consumption figures for AISH, and patient trust indices for AIPE. Operationalization is therefore the step that converts AI-HVF from a conceptual contribution into a usable evaluation framework.

This table serves three primary purposes. First, it functions as a rigorous evaluation tool, enabling researchers and policymakers to systematically assess the implementation and impact of AI in healthcare. Second, it offers a foundation for benchmarking, allowing comparison of diverse AI systems’ contributions toward healthcare value. Third, it promotes accountability by providing stakeholders with transparent, testable indicators to monitor progress effectively. Importantly, the inclusion of environmental, ethical, and trust-related metrics illustrates that the enhanced AI-Integrated AI-HVF extends beyond purely economic considerations to embrace the comprehensive spectrum of healthcare value. In this way, Table 2 provides the practical basis for testing, comparing, and refining the framework across settings.

#### Retrospective Stress Test of AI-HVF

A useful way to assess the practical utility of AI-HVF is to apply it retrospectively to a published AI deployment case, such as autonomous diabetic retinopathy screening in primary care. In the pivotal trial of an autonomous AI-based diagnostic system, the model demonstrated strong clinical performance, including 87.2% sensitivity, 90.7% specificity, and a 96.1% imageability rate, showing that AI can deliver specialist-level screening support in non-specialist settings [81,82].

Viewed through Porter’s value-based healthcare lens, this case would mainly emphasize improved outcomes relative to cost, such as earlier detection and potentially more efficient screening. However, when assessed through AI-HVF, the same case reveals additional value dimensions that are central to AI-enabled care but less visible in traditional value models, including explainability, workflow integration, governance, trust, fairness across patient groups, retraining and maintenance requirements, and long-term sustainability. For example, diabetic retinopathy AI deployment raises questions not only about diagnostic accuracy, but also about ungradable images, staff training, integration with clinical pathways, referral adherence, accountability for false negatives, and whether performance remains equitable across diverse populations [81,82,83,84].

This retrospective illustration suggests that AI-HVF can identify important strengths, gaps, and implementation burdens that are not fully captured by a narrower cost-outcome logic. For this reason, future research should apply AI-HVF retrospectively and prospectively across published AI clinical trials and real-world deployments to examine its discriminatory value relative to existing models and to refine its indicators in different healthcare settings.

### 4.5. Addressing Limitations in Traditional Models

The proposed framework addresses the limitations of traditional healthcare value models in five main ways.

1. First, it expands the structural dimension. In conventional models, structure is often limited to facilities, staff, and basic resources. In AI-HVF, structure also includes digital infrastructure, workforce readiness, and governance mechanisms such as ethics, privacy, and trust.
2. Second, it broadens the process dimension. Traditional frameworks focus mainly on diagnosis, treatment, and care delivery. AI-HVF extends process measurement to include algorithmic excellence in diagnosis, prediction, and personalization, as well as patient experience, preventive care, and explainability in AI-supported workflows.
3. Third, it redefines outcomes. Rather than focusing only on clinical endpoints, the framework includes trust in AI, equity, safety, care continuity, provider well-being, and environmental sustainability as important outcome dimensions.
4. Fourth, it introduces True Cost Assessment. The framework accounts not only for direct financial costs but also for governance, ethical oversight, workforce training, and environmental impact. This provides a more realistic view of the resources required for responsible AI implementation.
5. Fifth, it places governance at the center of the model. Governance functions as an overarching layer that shapes decisions across structure, process, outcomes, and costs. It ensures that fairness, privacy, accountability, and ethical responsibility remain embedded throughout the framework.

Taken together, these features make AI-HVF a more comprehensive and context-responsive model for AI-enabled healthcare. Its adaptive and cybernetic design also allows outcomes to feed back into structures and processes in real time, supporting continuous learning and improvement.

## 5. Comparative Landscape

AI-HVF complements, rather than duplicates, established digital health evaluation frameworks. The NASSS framework is particularly useful for understanding implementation complexity, nonadoption, and sustainability across the condition, technology, adopter, organization, and wider context, but it is not designed to provide a healthcare value account that explicitly integrates clinical excellence, equity, explainability, provider well-being, governance, and environmental sustainability as separate evaluative dimensions. Likewise, WHO digital health guidance offers an evidence-based lens for assessing benefits, harms, acceptability, feasibility, resource use, and equity, but it is oriented toward evaluating specific digital interventions rather than constructing a broader multidimensional value framework for AI-enabled healthcare systems. AI-HVF therefore extends the field by translating these implementation and evaluation concerns into a dedicated value architecture for AI in healthcare (see Table 3), with explicit attention to governance, fairness, trust, lifecycle cost, and sustainability [85,86].

## 6. Theoretical and Practical Implications

The AI-HVF represents a substantial advancement over traditional health-care value models by adding dynamic AI-specific components to the classical structure–process–outcome paradigm. We acknowledge that in the AI era, healthcare value is more than cost effectiveness or clinical outcomes. AI-HVF incorporates crucial dimensions such as algorithmic fairness, transparency, sustainability, and provider wellbeing as essential components of value creation. This framework redefines structure to include digital infrastructure and workforce readiness, and expands processes to include AI-augmented clinical excellence, patient experience, preventive care and explainability. Results are extended to include not just traditional clinical outcomes but also AI-related equity, safety, continuity and environmental impact. Also, the use of True Cost Assessment covers hidden and recurring AI costs, leading to a comprehensive evaluation. The AI-HVF institutionalizes governance, ethics and trust as an overarching shell. At its heart, the AI-HVF insists that accountability and fairness are prerequisites to realizing AI’s potential in health systems.

Importantly, the framework is also relevant to low-resource settings and low- and middle-income countries (LMICs) where AI adoption must be evaluated not only for technical sophistication, but also for affordability, scalability, and fit with local health system capacity. In such settings, AI-HVF can help determine if a solution strengthens essential service delivery, reduces workforce burden, improves access to underserved populations, and avoids deepening digital or geographic inequities. The framework could also facilitate context-specific evaluation of infrastructure constraints, such as limited connectivity, fragmented records, shortages of trained personnel, and uneven data quality. AI-HVF prioritizes implementation feasibility, in addition to outcomes, and can help inform more realistic and equitable decisions about which AI tools should be prioritized, adapted, or deferred in resource-constrained environments.

The framework is also well aligned with global health goals, including the Sustainable Development Goal 3 and the universal health coverage agenda more broadly. Its focus on equity, access, quality, safety, prevention and sustainability reinforces commitments to SDG 3 for healthy lives, reduced mortality and improved health system performance. At the same time, its emphasis on equitable distribution of resources, continuity of care and responsible governance is consistent with the principles of universal health coverage that seek to ensure access to needed services without financial hardship. In this sense, AI-HVF extends beyond the evaluation of the technological performance to a tool to determine if AI is truly contributing to resilient, inclusive and people-centred health systems.

Practically, AI-HVF provides a comprehensive and flexible tool for researchers, policy makers and healthcare stakeholders to assess and compare the performance of AI implementations based on multidimensional value indicators. A phased pilot implementation strategy is suggested to allow empirical testing. Second, a small number of AI use cases should be selected from a variety of clinical settings, including at least one low-resource or LMIC setting, where feasibility and equity concerns are particularly salient. Second, each AI-HVF domain should be translated into measurable indicators developed through stakeholder consultation and taking into consideration local priorities and data infrastructure available. Third, baseline and follow-up assessments should be made to compare across domains of structure, process, outcome, cost and governance. Fourth, qualitative feedback from patients, clinicians, managers and policymakers should be used to assess the usability, trust and unintended consequences of the tool. Finally, the pilot should be used to refine the framework, validate the relevance of the indicators and determine whether AI-HVF can support routine monitoring, cross-site comparison and continuous learning. Such a stepwise approach would help the framework move from a conceptual proposal to a practical evaluation tool.

The framework captures real-time feedback loops to model healthcare as a continuously learning system that integrates technological advances with patient-centric and societal goals. This framework will help to guide the ethical deployment of AI, promote provider well-being, and encourage sustainable practices while also addressing the challenges of equity and inclusion that AI’s complexity presents. Operationalizing abstract concepts of value in concrete metrics turns them into actionable data, thereby contributing to transparency and accountability. AI-HVF ultimately prepares healthcare systems to responsibly harness AI-enabled innovation, so that progress translates into fair, trustworthy, and ecologically sustainable care that meets the changing needs of patients and providers.

The adoption of the AI-Augmented Healthcare Value Framework has important implications for health system leaders and policymakers. Organizations can move beyond static metrics and episodic evaluation by embedding dynamic, multidimensional measures of value such as equity, safety, sustainability, and trust into their operational fabric of healthcare delivery. This allows for more nimble decision-making, forward-looking risk management, and the responsible use of AI technologies. Most importantly, it enables the development of transparent benchmarking tools and standardized measurement systems to promote cross-organizational learning and ethical AI adoption. The implementation of this framework in real-world settings allows healthcare practitioners to attain more patient-centric, equitable, and sustainable results, aligning technological advancement with fundamental societal values.

The framework is designed to be adaptable across healthcare contexts. Its core dimensions remain stable, but the weighting and operational indicators should differ by setting: prevention and continuity in primary care, diagnostic precision and safety in specialized care, rapid workflow support in acute care, and adherence and long-term engagement in chronic care. Successful implementation of AI-HVF will depend on organizational readiness, including clinician engagement, leadership support, workflow redesign, digital infrastructure, interoperability, and resources for training and maintenance. These issues do not invalidate the framework; rather, they define the conditions under which the framework can be meaningfully applied and evaluated in practice.

## 7. Future Research Directions

The AI-HVF provides a multidimensional framework to evaluate the clinical, ethical, operational, and sustainability impact of AI in healthcare. Its real value, however, will depend on empirical validation and iterative refinement. In the short run, future research should focus on validation studies testing feasibility, reliability and sensitivity to change of the proposed indicators (e.g., AICE, AIPE, AIEI, AISH, AITEG) in different clinical settings. These dimensions need to be tested in large-scale multisite studies to determine whether they result in measurable improvements in patient outcomes, provider experience, equity, safety, and system resilience, as well as to identify implementation barriers and constraints in specific contexts.

A second short-term priority is the creation of standardized metrics and benchmarking systems. The framework talks about concepts such as algorithmic fairness, trust and environmental sustainability, but these need to be translated into validated and comparable tools. Development of cross-setting metric should take into account international standards and reporting approaches such as sustainability accounting methodologies (e.g., Greenhouse Gas Protocol) and health-system sustainability indices (e.g., NHS Digital Sustainability Index), so that environmental performance can be measured consistently and meaningfully.

In the longer term, research should focus on policy integration, governance and system level adoption. Comparative analyses are needed to assess how AI-HVF might be integrated into regulatory frameworks, procurement policies, accreditation systems, and health system performance monitoring. Work in the longer term should also examine whether the framework is able to support ongoing quality improvement, responsible AI governance, and alignment with wider health system goals. Policy-oriented studies should examine the prospects for adaptation of AI-HVF across countries and health systems that vary in digital maturity, especially in low-resource settings and LMICs.

The study of human–AI interactions and sociotechnical dynamics is also relevant. AI systems will inevitably change clinical workflows, decision-making authority, and patient–provider relationships. Future research should use mixed-methods design to investigate the progression, challenge, and erosion of trust in AI recommendations over time, and the impact of transparency, explainability, and usability on adoption. The studies should be conducted in an interdisciplinary way involving informaticians, clinicians, ethicists, data scientists, health services researchers and policymakers, in such a way that technical performance and ethical legitimacy are considered together.

The framework also points to the need for more robust ethics and governance systems. In future work, governance should be implemented via fairness audits, privacy safeguards, accountability mechanisms, and compliance pathways that balance innovation with oversight. Ethical impact assessments and comparative policy studies across regions can identify practical governance models that are context-sensitive and scalable.

Another major research frontier is sustainability. Studies should measure the environmental impact of AI using life-cycle assessment and carbon accounting methods, and assess the effect of digital health interventions on energy usage, emissions, and resource consumption. This work helps decision makers choose AI systems that are not only effective but also environmentally responsible by linking clinical benefit to environmental cost.

Finally, future research should further examine the role of AI in population health, learning health systems, and equity. Researchers should explore how AI can help predictive epidemiology, preventive care, health education, and more equitable distribution of resources, and explore feedback loops that enable continuous learning and improvement. It will be important to ensure that data sourcing, stakeholder participation, and implementation design are inclusive to build AI systems that respond to diverse populations and align with long-term health equity goals.

Although AI-HVF is intended to be practical and implementation-oriented, the present paper remains a conceptual development study rather than a field evaluation. Accordingly, detailed analysis of clinician resistance, implementation costs, organizational change management, and technical infrastructure readiness was beyond the scope of this manuscript. These factors are nevertheless recognized as essential determinants of successful adoption, and future empirical work will assess how AI-HVF performs under real-world organizational, financial, and technological constraints.

## 8. Conclusions

The AI-Augmented Healthcare Value Framework (AI-HVF) provides a multidimensional structure for evaluating AI-enabled healthcare across clinical, operational, ethical, societal, cost, and governance domains. By linking structure, process, outcome, cost, and governance through adaptive feedback loops, the framework is intended to support more context-sensitive assessment of how AI systems affect care quality, equity, safety, continuity, provider well-being, sustainability, and trust over time.

Rather than treating outcomes as fixed endpoints, AI-HVF conceptualizes them as signals that can reshape infrastructure, workflows, oversight, and future implementation decisions. In this way, the framework is designed not only to assess whether AI performs effectively, but also to examine whether it is implemented responsibly, maintained safely, and aligned with broader health-system goals and public value.

At the same time, the present study remains conceptual and does not claim universal empirical validation across all healthcare settings. The framework is therefore best understood as an evaluative foundation that now requires retrospective case application, prospective pilot testing, and indicator refinement across diverse clinical and resource environments, including lower-resource systems where feasibility, affordability, and governance constraints may be especially important.

Overall, AI-HVF offers a structured basis for benchmarking, implementation planning, and continuous learning in AI-enabled healthcare. Its practical value will depend on how effectively its dimensions are operationalized, adapted to local context, and tested in real-world use cases to support ethical, equitable, and sustainable AI integration in health systems.

## Figures and Tables

**Table 1:** Comparison of traditional healthcare value models.

Model	Traditional Focus	Why It Falls Behind in the AI Era	Potential AI Adaptations
Donabedian’s Structure–Process–Outcome	Linear sequence from structure to process to outcomes, with emphasis on clinical quality, safety, and satisfaction.	It is too linear to capture AI feedback loops. It also does not account for algorithms, cloud infrastructure, governance, or AI-related issues such as fairness, transparency, and trust.	Expand structure to include digital infrastructure, data governance, and algorithmic systems; expand outcomes to include fairness, explainability, and trust.
Porter’s Value-Based Healthcare	Improve patient outcomes relative to cost and increase efficiency in care delivery.	It does not fully capture hidden AI costs such as infrastructure, retraining, ethics review, and cybersecurity. It also overlooks trust, explainability, and provider workload, and may miss inequities caused by biased data.	Broaden cost accounting to include AI lifecycle and governance costs; broaden outcomes to include patient trust, explainability, and equity.
Quadruple Aim	Improve patient experience, population health, cost, and provider well-being.	It does not address algorithmic governance, bias, or explainability. It also does not capture the environmental costs of AI or the new workload pressures AI may create.	Add AI governance, fairness monitoring, and sustainability metrics; include measures of digital workload, alert burden, and environmental impact.

**Table 2:** Dimensions in AI-HVF: definitions, descriptions, and metrics.

Dimension	Definition	Description	Example Metrics
AICE—AI-Supported Clinical Excellence	The use of AI to improve accuracy, timeliness, and personalization of diagnosis and treatment.	AI-driven decision support, predictive analytics, and precision medicine enable clinicians to deliver evidence-based, tailored care.	Diagnostic sensitivity/specificity; reduction in diagnostic errors; % patients receiving AI-personalized treatments.
AIPE—AI-Enhanced Patient Experience	Application of AI to improve communication, empathy, and convenience in patient interactions.	Chatbots, multilingual support, and personalized engagement enhance satisfaction while reducing perceived barriers.	Patient-reported satisfaction scores (PREMs); trust ratings in AI-assisted care; chatbot adoption rates.
AIOE—AI-Enabled Operational Efficiency	Leveraging AI to optimize healthcare resource allocation and workflows.	Automates scheduling, supply chain management, and administrative tasks to reduce waste and waiting times.	Reduction in avg. wait times; scheduling accuracy; documentation time per patient; cost savings.
AIPPH—AI-Driven Preventive & Population Health	Use of AI for proactive disease prevention and population-level health monitoring.	Predictive analytics and remote monitoring to detect risks early, reduce hospitalizations, and inform public health decisions.	Early disease detection rate; avoided hospitalizations; epidemic prediction accuracy; % population covered by monitoring.
AISCR—AI-Supported Care Continuity & Retention	AI tools that maintain consistent, long-term care relationships and follow-up.	Facilitates adherence reminders, chronic disease tracking, and post-discharge monitoring to prevent fragmentation.	Readmission rates; medication adherence; % patients receiving AI follow-up; continuity of care index.
AIEI—AI-Enabled Equity & Inclusion	Ensuring fairness and inclusivity in AI design and deployment.	Uses representative datasets, bias audits, and culturally sensitive AI solutions to reduce disparities.	Fairness audit scores with subgroup sensitivity/specificity gap, calibration gap, equal opportunity difference; variance in outcomes across demographics; % of minority data in training sets; Access coverage or reach of AI interventions; Participation rate in co-design or participatory design processes; Community engagement or stakeholder consultation score.
AISR—AI-Integrated Safety & Reliability	Protecting patients from errors or failures of AI systems.	Continuous performance monitoring, model drift detection, and adversarial robustness testing ensure safety.	AI-related adverse event rates; model drift incidents detected; % explainability compliance.
AIPW—AI-Supported Provider Well-Being	AI designed to reduce clinician burden and burnout.	Automates documentation and repetitive tasks, prevents alert fatigue, and optimizes workflows for well-being.	Burnout survey scores (e.g., Maslach Index); after-hours workload; documentation time saved.
AISH—AI-Driven Sustainability & Green Health	Reducing environmental impact of AI and healthcare operations.	Monitors and minimizes energy consumption and carbon footprint of AI technologies.	kWh per AI model run; CO_2_ emissions per operation; % adoption of green data centers.
AITE—AI-Enabled Transparency & Explainability	Providing interpretable, understandable AI outputs for patients and clinicians.	Enhances trust through human-interpretable explanations of AI recommendations.	% AI systems with explanation interfaces; clinician trust survey results; explanation completeness index.
AILH—AI-Powered Learning Health Systems	Embedding continuous learning into healthcare through AI.	Uses real-world data and iterative updates to refine protocols and improve outcomes.	Frequency of AI model updates; time-to-integrate new evidence; % clinical guidelines updated with AI insights.
Societal Value	The contribution of healthcare to public health, social resilience, and connectedness.	AI supports vaccination campaigns, health education, and community-level equity.	Public health outcomes improved; vaccination rates supported by AI; community health equity index.
Cost+ (True Cost Assessment)	Full life-cycle cost of AI implementation and maintenance, including compute, integration, training, audits, monitoring, updating, and workflow disruption. Comprehensive assessment of financial, ethical, and environmental costs of AI.	Includes infrastructure, governance, training, opportunity costs, and environmental impacts.	Total cost of ownership; cost per use case; monitoring and retraining cost; workflow disruption cost; cost per outcome gained.

**Table 3:** Positioning AI-HVF Against NASSS and WHO Digital Health Guidance.

Figure	Main Focus	What It Captures Well	What AI-HVF Adds
NASSS	Non-adoption, abandonment, scale-up, spread, sustainability, and adaptation over time.	Implementation complexity, sociotechnical fit, and longitudinal change.	Explicit value dimensions such as equity, explainability, trust, provider well-being, and environmental sustainability.
WHO digital health guidance	Benefits, harms, acceptability, feasibility, resource use, and equity.	Evidence-informed evaluation of digital interventions and implementation considerations.	A broader AI-specific value structure that integrates governance, fairness, lifecycle cost, and operationalized healthcare value.
AI-HVF	Value in AI-enabled healthcare across structure, process, outcome, cost, and governance.	Cross-layer assessment of clinical, organizational, ethical, and societal value.	Designed specifically for AI-enabled healthcare systems and their unique risks and benefits.

## Data Availability

The original contributions presented in this study are included in the article. Further inquiries can be directed to the corresponding author.
